# Causal Association Between Commonly Used Medicines and Diabetes‐Related Eye Diseases: Univariable and Multivariable Mendelian Randomization Study

**DOI:** 10.1155/jdr/6075025

**Published:** 2026-04-29

**Authors:** Yu Wang, Chak Kwong Cheng, Lamusi A., Mingyue Jin, Chang Wang

**Affiliations:** ^1^ Department of Endocrinology and Metabolism, Shenzhen University General Hospital, Shenzhen University, Shenzhen, 518055, China, szu.edu.cn; ^2^ Department of Pharmacology, Max Planck Institute for Heart and Lung Research, Bad Nauheim, 61231, Germany, mpi-hlr.de; ^3^ Department of Ophthalmology, International Mongolian Hospital of Inner Mongolia, Hohhot, 010020, China; ^4^ Department of Nephrology, Institute of Nephrology, Second Affiliated Hospital of Hainan Medical University, Haikou, 570311, China, hainmc.edu.cn

**Keywords:** adrenergics, calcium channel blockers, diabetic maculopathy, diabetic retinopathy, instrumental variables, mendelian randomization

## Abstract

**Background:**

Diabetic patients require long‐term polypharmacy, yet the causal effects of these medications on ocular complications—specifically diabetic retinopathy (DR), diabetic maculopathy (DMac), and glaucoma—remain unclear due to confounding in observational studies.

**Methods:**

We performed a Mendelian randomization (MR) study using GWAS summary statistics for 23 common medication classes and five diabetic eye diseases. Multivariable MR (MVMR) was employed to adjust for key comorbidities (e.g., hypertension, type 2 diabetes), while enrichment analyses explored biological mechanisms.

**Results:**

In the univariable MR (UVMR) analysis, drugs used in diabetes, agents acting on the renin–angiotensin system, and several other medication classes showed significant associations with increased risks of diabetic eye diseases. Crucially, MVMR confirmed robust causal links after adjusting for key comorbidities. Specifically, drugs used in diabetes remained associated with DMac (OR = 1.44, *p* = 1.31 × 10^−11^), DR (OR = 1.23, *p* = 1.26 × 10^−8^), neovascular glaucoma (OR = 1.19, *p* = 0.003), and senile cataract (OR = 1.10, *p* = 9.25 × 10^−12^) independent of type 2 diabetes liability. Similarly, thyroid preparations retained significance for DMac (OR = 1.26, *p* = 4.35 × 10^−10^), DR (OR = 1.17, *p* = 9.53 × 10^−10^), and senile cataract (OR = 1.04, *p* = 0.004) after adjusting for hypothyroidism. Additionally, adrenergic inhalants were independently linked to senile cataract (OR = 1.07, *p* = 0.008) after adjusting for asthma. Pathway analysis highlighted hormone transport and MAPK signaling as potential mechanisms.

**Conclusion:**

Our findings provide genetic evidence supporting potential comorbidity‐independent causal links between specific systemic medications—particularly diabetes and thyroid drugs—and ocular complications, suggesting the importance of ophthalmological monitoring in these patients.

## 1. Introduction

According to data from the International Diabetes Federation, the global prevalence of diabetes is projected to exceed 700 million by 2045, representing ~10% of the world’s population [1]. Research indicates that half of all diabetes patients will develop diabetic microvascular complications, including ocular diseases such as diabetic retinopathy (DR) and diabetic maculopathy (DMac) [2]. Additionally, neovascular glaucoma (NVG) and cataracts constitute severe ocular complications of diabetes [3, 4]. Among type 2 diabetes patients alone, the prevalence of DR ranges from 12.2% to 61.0% in developing countries and 9.9% to 48.1% in developed nations [5]. This imposes a substantial psychological and economic burden. Genetic predisposition and lifestyle factors play pivotal roles in the progression of pathological changes associated with DR [6, 7]. However, most established factors lack accuracy in predicting the onset of diabetic eye disease. Therefore, identifying more reliable risk factors for the recognition and early prediction of diabetic eye disease is of paramount importance.

Not only can medication use mitigate the progression of diabetic eye disease, but certain medication uses may cause or exacerbate disease progression. For example, antivascular endothelial growth factor (anti‐VEGF) injections (including drugs such as ranibizumab, bevacizumab, and aflibercept) are currently the primary treatment for all stages of DR and DMac [8, 9]. However, peroxisome proliferator‐activated receptor gamma agonists may exacerbate diabetic macular edema, while semaglutide (a GLP‐1 receptor agonist) may slightly worsen DR [10]. Furthermore, results from randomized clinical trials indicate that faricimab demonstrates superior improvement in central subfoveal thickness compared with other anti‐VEGF therapies when administered at extended dosing intervals [11]. Antiglaucoma medications include carbonic anhydrase inhibitors (oral and topical), beta‐blockers, and alpha‐2 agonists. These agents reduce aqueous humor production but may increase inflammation and exacerbate adhesive angle closure [3]. Additionally, carnosine may effectively prevent and treat age‐related cataracts through its antioxidant and antiglycation properties [12]. It is worth noting that even if these drugs show some effect on the disease, these observational studies may be limited by sample size and potential confounding factors. Indicative confounding and reverse causation are particularly salient issues in pharmacoepidemiology, in addition to traditional confounders. For example, patients with more severe disease may be more likely to use certain drugs, or drug use may be a consequence rather than a cause of disease progression. Therefore, we are interested in evaluating the potential of common medications as targets for preventive interventions in diabetic eye disease and investigating their etiologic role in diabetic eye disease.

Mendelian randomization (MR) is a robust method based on Mendel’s first and second laws of genetic inheritance: the law of segregation and the law of independent assortment [13]. Most MR studies use genetic variants significantly associated with exposure as an instrumental variable (IV) to assess the association between genetically predicted exposure and outcome [14]. Because genetic variants are randomly inherited from parents to offspring at conception, they are unlikely to be affected by potential confounders and reverse causation [14]. MR‐based study designs allow for the investigation of many exposures that are not amenable to randomized controlled trial (RCT) studies [15]. Overall, MR studies can effectively overcome the indicative confounding and reverse causality bias common in observational studies by utilizing genetic variation as IVs. For example, Wang et al. [16] suggested that thiazide diuretics may reduce the risk of hepatocellular carcinoma in Europeans and East Asians, whereas β‐adrenergic receptor blockers may increase the risk of hepatocellular carcinoma, especially in Europeans. However, the relationship between commonly used medicines and diabetic eye diseases using the MR method has not yet been investigated.

In this study, we applied a comprehensive framework integrating univariable and multivariable MR (MVMR) with post‐GWAS functional annotation. We aimed to systematically evaluate the potential causal associations between commonly used medications and diabetic eye diseases (including DR, DMac, NVG, senile cataract, and POAG) and to further prioritize robust signals using gene mapping and pathway enrichment analyses. By leveraging genetic instruments to mitigate confounding and reverse causation inherent in observational studies, our approach seeks to generate mechanistically informed hypotheses regarding the role of systemic pharmacotherapy in ocular complications.

## 2. Materials and Methods

### 2.1. Study Framework

**Figure 1:**
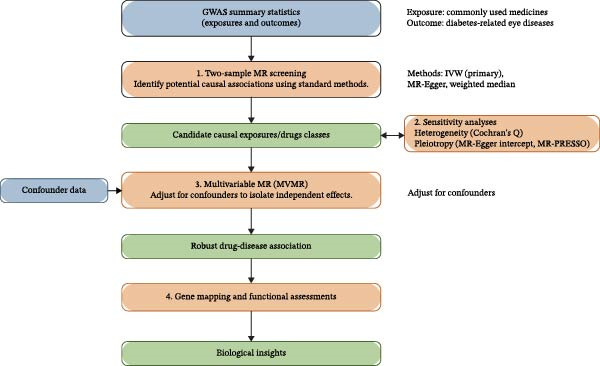
Overview of Mendelian randomization.

This study employed a two‐sample MR design to systematically investigate the potential causal relationships between commonly used medications and the risk of diabetic eye diseases. In the first stage, univariable MR (UVMR) was conducted to assess the total effect of each of the 23 commonly used medications. In the second stage, for associations showing nominal significance in the UVMR analysis, MVMR was performed to estimate effects independent of key comorbidities (e.g., hypertension, type 2 diabetes) rather than the eye diseases themselves. Subsequently, drug categories and biological pathways were screened through gene mapping and gene enrichment analysis. This MR analysis was conducted in accordance with the *STROBE-MR* [17]. The analytical framework was built upon three fundamental IV assumptions [18]: (a) the IVs are strongly associated with the exposure of interest; (b) the IVs are not related to confounding factors that may influence the outcome; and (c) the IVs affect the outcome exclusively through the exposure pathway. A schematic overview of the overall workflow is illustrated in Figure 1. Since all the GWAS summary statistics used were publicly accessible, no additional ethical approval or informed consent was required for this analysis.

### 2.2. Data Sources

We applied a two‐sample MR design to explore potential causal links between the use of common medications and the risk of diabetic eye diseases. Twenty‐three frequently prescribed medications were considered as exposures, and several ocular conditions associated with diabetes were treated as outcomes.

Summary‐level GWAS data for DR (14,142 cases and 82,287 controls), DMac (4,603 cases and 82,287 controls), neovascular glaucoma (NVG; 1,494 cases and 485,314 controls), primary open‐angle glaucoma (POAG; 10,832 cases and 473,757 controls), and senile cataract (83,886 cases and 409,535 controls) were extracted from the FinnGen R12 release, which includes individuals of European descent. GWAS datasets for the 23 medication exposures were retrieved from the OpenGWAS database, also restricted to populations of European ancestry [19]. Comprehensive details of all included GWAS datasets, including sample sizes, ancestry, and data sources, are summarized in Supporting Information 1: Table S1. Additionally, summary statistics for the confounding factors used in the MVMR analysis (e.g., type 2 diabetes, hypertension, asthma/COPD, and glaucoma) were primarily obtained from the Million Veteran Program (MVP) and other large‐scale consortia, as detailed in Supporting Information 2: Table S8.

### 2.3. IV Selection

Single nucleotide polymorphisms (SNPs) associated with each exposure were selected as IVs for the MR analysis. The SNP selection process was carried out as follows:

First, SNPs significantly associated with each medication class were selected as potential IVs. For UVMR, SNPs showing genome‐wide significance for the exposure traits (*p*  < 5 × 10^−6^) were identified from previous GWAS datasets [20, 21]. This approach was chosen because the conventional genome‐wide significance threshold (*p*  < 5 × 10^−8^) yielded an insufficient number of IVs for a robust analysis, a common strategy employed in MR studies when dealing with exposure GWAS with limited statistical power. For MVMR, a more stringent threshold of *p*  < 5 × 10^−8^ was used for both the primary exposure and the covariates [22, 23]. To ensure adequate allele frequency representation, only variants with a minor allele frequency (MAF) greater than 0.01 were retained [24]. Next, linkage disequilibrium (LD) pruning was performed to eliminate correlated SNPs, applying a threshold of *r* ^2^ < 0.001 within a 10,000 kb window [25]. When an IV was absent in the outcome GWAS dataset, a highly correlated proxy SNP (*r* ^2^ > 0.8) was identified and substituted for the missing variant [26]. The strength of each IV was evaluated using the *F*‐statistic to avoid weak‐instrument bias, calculated as *F = R* ^2^ × (*N − 2*) */* (1 − *R* ^2^), where *R^2^* represents the proportion of variance in the exposure explained by the SNP, and *N* denotes the corresponding sample size. IVs with *F*‐values greater than 10 were considered sufficiently strong [27].

### 2.4. MR Analysis

The primary causal estimates between the use of common medications and diabetic eye diseases were obtained using the inverse variance weighted (IVW) method, which provides the most efficient estimates when all IVs are valid [28]. To confirm the consistency and robustness of the findings, three complementary MR approaches were also implemented: MR‐Egger regression, weighted median (WM), and weighted mode analyses [29]. MR‐Egger regression accounts for potential directional pleiotropy through the intercept term and yields unbiased estimates when pleiotropic effects are present. A significant MR‐Egger intercept (*p*  < 0.05) suggests directional pleiotropy and possible bias in IVW estimates [28, 30]. The WM estimator, by contrast, remains consistent even when up to 50% of the IVs are invalid, while the weighted mode method identifies the most frequent causal estimate among valid instruments [31]. All MR procedures were conducted using the *TwoSampleMR* package implemented in R software (version 4.0.5). Results were summarized as odds ratios (ORs) with 95% confidence intervals (CIs) and visualized through scatter and forest plots. Statistical significance was set at *p*  < 0.05. To account for multiple testing, *p*‐values were adjusted using the false discovery rate (FDR) method, with significance defined at P‐FDR < 0.05 [32].

### 2.5. Sensitivity Analysis

We performed a series of sensitivity analyses to identify possible heterogeneity and horizontal pleiotropy in the MR estimates. Heterogeneity across IVs was evaluated using Cochran’s *Q* test; a nonsignificant result (*p*  > 0.05) indicated low heterogeneity and suggested that the IVs were largely consistent, having little impact on the overall IVW estimates [33]. Potential horizontal pleiotropy was further examined using MR‐Egger regression, which tests whether the average pleiotropic effect deviates from zero through the intercept term [34, 35]. An intercept value close to zero or statistically nonsignificant was interpreted as evidence against directional pleiotropy. To further correct for pleiotropic bias, the MR‐PRESSO approach was applied to detect and exclude outlier SNPs (*p*  < 0.05), after which causal estimates were recalculated [35]. Additionally, funnel plots were used to visualize the distribution of SNP effects, and leave‐one‐out analyses were conducted to confirm the stability of the results.

### 2.6. MVMR

To assess the association between key commonly used medications and diabetic eye diseases independent of confounding factors, this study conducted an MVMR analysis. This method estimates the direct effect of the exposure on the outcome while adjusting for the genetically predicted levels of covariates, including hypertension, type 2 diabetes, asthma/COPD, glaucoma, hyperlipidemia, and hypothyroidism. We selected SNPs significantly associated (*p*  < 5 × 10^−8^) with either the exposures or the confounding traits. To ensure independence, we performed LD clumping (*r* ^2^ < 0.001, window size = 10,000 kb) separately for exposure and confounder SNPs, merged the valid instruments, and removed duplicates. A second round of LD clumping was applied to the combined set to eliminate residual correlation. Finally, we harmonized the data by correcting for strand orientation and excluding palindromic SNPs with intermediate allele frequencies to ensure the validity of the genetic instruments used in the multivariable framework [36].

### 2.7. Gene Mapping

Combining the UVMR and MVMR results, we focused on key medications showing robust associations, specifically drugs used in diabetes and agents acting on the renin–angiotensin system [37]. We screened IVs/SNPs separately and utilized the R package “biomaRt” for biological functional annotation. The specific steps were as follows: first, we remotely connected to the Ensembl Variation Database via the biomaRt interface and selected the human reference genome dataset (hsapiens_snp, version GRCh38.p14). Next, using the list of SNP reference sequence identifiers (rsIDs) as the input vector, we performed automated batch retrieval using the getBM function. Within the retrieval model, the filter tag was set to snp_filter to extract key attributes, including the unique rsID, chromosomal location (chr_name, chrom_start), and the associated gene symbol (associated_gene). This process constructed a one‐to‐one mapping table between SNPs and target genes, providing foundational data for subsequent functional enrichment analyses.

### 2.8. Functional Enrichment Analysis

Functional enrichment of the analyzed gene sets was performed using the R package “clusterProfiler.” For Gene Ontology (GO) enrichment analysis, three subcategories were analyzed: biological process (BP), molecular function (MF), and cellular component (CC) [38]. Additionally, Kyoto Encyclopedia of Genes and Genomes (KEGG) pathway enrichment analysis was performed, excluding human disease pathways to focus on mechanistic signaling [39]. The *p*‐values for the significance of the enriched pathways were corrected using the Benjamini–Hochberg (BH) method, and the corrected *p*‐value (*p*.adjust < 0.05) was used as the screening criterion for identifying the top significant pathways.

## 3. Results

### 3.1. IVs Selection

In this study, 749 IVs associated with 23 commonly used medicines were identified. The *F*‐statistics for all IVs were greater than 10 (range 29.70–897.13), indicating a low risk of weak‐instrument bias. Detailed information on the selected IVs is provided in Supporting Information 1: Table S2. All identified SNPs were successfully matched with the outcome GWAS datasets, and proxy SNPs were used where necessary (Supporting Information 1: Table S3).

### 3.2. UVMR: Associations of Commonly Used Medicines and Diabetic Eye Disease

We initially assessed the total causal effects of 23 medication classes on diabetic eye diseases using the IVW method. After removing outliers identified by MR‐PRESSO (Supporting Information 1: Table S4, Table 1) and ensuring no heterogeneity and absence of horizontal pleiotropy (Table 2), several medication classes showed potential associations. However, to control for false positives due to multiple testing, we applied FDR correction.

**Table 1:** MR‐PRESSO analysis for outlier detection and correction.

Exposure	Outcome	Raw		Outlier corrected		Global *P*	Number of outliers	Distortion *P*
		OR (CI%)	*P*	OR (CI%)	*P*			
Adrenergics, inhalants	Diabetic maculopathy	0.97 (0.88–1.08)	0.62	NA	NA	0.009	0	NA
Adrenergics, inhalants	Diabetic retinopathy	1.01 (0.95–1.07)	0.74	NA	NA	0.075	0	NA
Adrenergics, inhalants	Neovascular glaucoma	1.06 (0.92–1.22)	0.41	NA	NA	0.464	0	NA
Adrenergics, inhalants	Primary open‐angle glaucoma	0.97 (0.91–1.03)	0.28	NA	NA	0.103	0	NA
Adrenergics, inhalants	Senile cataract	1.12 (1.08–1.15)	0	NA	NA	0.086	0	NA
Agents acting on the renin–angiotensin system	Diabetic maculopathy	0.97 (0.91–1.05)	0.47	NA	NA	0.694	0	NA
Agents acting on the renin–angiotensin system	Diabetic retinopathy	0.98 (0.93–1.03)	0.4	NA	NA	0.014	0	NA
Agents acting on the renin–angiotensin system	Neovascular glaucoma	1.09 (0.96–1.24)	0.16	NA	NA	0.474	0	NA
Agents acting on the renin–angiotensin system	Primary open‐angle glaucoma	1 (0.95–1.06)	0.94	NA	NA	0.007	0	NA
Agents acting on the renin–angiotensin system	Senile cataract	1.08 (1.05–1.11)	0	NA	NA	0.069	0	NA
Anilides	Diabetic maculopathy	1.04 (0.85–1.28)	0.71	NA	NA	0.947	0	NA
Anilides	Diabetic retinopathy	1.25 (0.97–1.61)	0.15	NA	NA	0.477	0	NA
Anilides	Neovascular glaucoma	1.03 (0.49–2.17)	0.94	NA	NA	0.261	0	NA
Anilides	Primary open‐angle glaucoma	0.94 (0.77–1.16)	0.61	NA	NA	0.694	0	NA
Anilides	Senile cataract	1.11 (0.96–1.27)	0.2	NA	NA	0.245	0	NA
Antiglaucoma preparations and miotics	Diabetic maculopathy	0.97 (0.91–1.03)	0.36	NA	NA	0.793	0	NA
Antiglaucoma preparations and miotics	Diabetic retinopathy	1.01 (0.97–1.06)	0.57	NA	NA	0.36	0	NA
Antiglaucoma preparations and miotics	Neovascular glaucoma	1.11 (0.98–1.25)	0.13	NA	NA	0.543	0	NA
Antiglaucoma preparations and miotics	Primary open‐angle glaucoma	1.84 (1.7–2)	0	NA	NA	0.341	0	NA
Antiglaucoma preparations and miotics	Senile cataract	1.04 (1.02–1.07)	0.01	NA	NA	0.349	0	NA
Antihistamines for systemic use	Diabetic maculopathy	0.96 (0.69–1.33)	0.82	NA	NA	0.313	0	NA
Antihistamines for systemic use	Diabetic retinopathy	1.02 (0.88–1.2)	0.77	NA	NA	0.561	0	NA
Antihistamines for systemic use	Neovascular glaucoma	1.51 (1.04–2.2)	0.07	NA	NA	0.324	0	NA
Antihistamines for systemic use	Primary open‐angle glaucoma	1.15 (0.96–1.37)	0.18	NA	NA	0.091	0	NA
Antihistamines for systemic use	Senile cataract	1.1 (1.02–1.2)	0.05	NA	NA	0.088	0	NA
Antiinflammatory and antirheumatic products, nonsteroids	Diabetic maculopathy	1.27 (0.91–1.78)	0.24	NA	NA	0.662	0	NA
Antiinflammatory and antirheumatic products, nonsteroids	Diabetic retinopathy	1.09 (0.96–1.23)	0.27	NA	NA	0.932	0	NA
Antiinflammatory and antirheumatic products, nonsteroids	Neovascular glaucoma	1.47 (0.8–2.69)	0.26	NA	NA	0.591	0	NA
Antiinflammatory and antirheumatic products, nonsteroids	Primary open‐angle glaucoma	1.41 (1.02–1.94)	0.09	NA	NA	0.271	0	NA
Antiinflammatory and antirheumatic products, nonsteroids	Senile cataract	1.02 (0.88–1.18)	0.84	NA	NA	0.431	0	NA
Antimigraine preparations	Diabetic maculopathy	1.04 (0.94–1.14)	0.45	NA	NA	0.342	0	NA
Antimigraine preparations	Diabetic retinopathy	1.02 (0.98–1.07)	0.37	NA	NA	0.822	0	NA
Antimigraine preparations	Neovascular glaucoma	1.04 (0.91–1.18)	0.57	NA	NA	0.722	0	NA
Antimigraine preparations	Primary open‐angle glaucoma	1.06 (1–1.12)	0.1	NA	NA	0.378	0	NA
Antimigraine preparations	Senile cataract	1.01 (0.98–1.04)	0.6	NA	NA	0.371	0	NA
Antithrombotic agents	Diabetic maculopathy	0.85 (0.66–1.11)	0.26	NA	NA	0.324	0	NA
Antithrombotic agents	Diabetic retinopathy	0.93 (0.78–1.1)	0.42	NA	NA	0.204	0	NA
Antithrombotic agents	Neovascular glaucoma	0.74 (0.48–1.13)	0.19	NA	NA	0.434	0	NA
Antithrombotic agents	Primary open‐angle glaucoma	0.88 (0.7–1.1)	0.28	NA	NA	0.156	0	NA
Antithrombotic agents	Senile cataract	1.04 (0.93–1.16)	0.5	NA	NA	0.055	0	NA
Beta blocking agents	Diabetic maculopathy	1 (0.9–1.11)	0.97	NA	NA	0.428	0	NA
Beta blocking agents	Diabetic retinopathy	1.04 (0.97–1.11)	0.3	NA	NA	0.119	0	NA
Beta blocking agents	Neovascular glaucoma	1.02 (0.86–1.2)	0.83	NA	NA	0.694	0	NA
Beta blocking agents	Primary open‐angle glaucoma	1.09 (1.01–1.18)	0.03	NA	NA	0.186	0	NA
Beta blocking agents	Senile cataract	1.08 (1.04–1.12)	0	NA	NA	0.228	0	NA
Calcium channel blockers	Diabetic maculopathy	0.98 (0.91–1.06)	0.63	NA	NA	0.241	0	NA
Calcium channel blockers	Diabetic retinopathy	1 (0.95–1.05)	0.92	NA	NA	0.08	0	NA
Calcium channel blockers	Neovascular glaucoma	1.14 (1–1.3)	0.05	NA	NA	0.285	0	NA
Calcium channel blockers	Primary open‐angle glaucoma	1.01 (0.95–1.08)	0.69	NA	NA	0.001	0	NA
Calcium channel blockers	Senile cataract	1.08 (1.05–1.11)	0	NA	NA	0.007	0	NA
Diuretics	Diabetic maculopathy	0.98 (0.91–1.06)	0.69	NA	NA	0.625	0	NA
Diuretics	Diabetic retinopathy	1.01 (0.96–1.06)	0.69	NA	NA	0.072	0	NA
Diuretics	Neovascular glaucoma	1.04 (0.91–1.19)	0.58	NA	NA	0.386	0	NA
Diuretics	Primary open‐angle glaucoma	1.05 (0.99–1.11)	0.12	NA	NA	0.015	0	NA
Diuretics	Senile cataract	1.04 (1.01–1.07)	0.01	NA	NA	0.057	0	NA
Drugs affecting bone structure and mineralization	Diabetic maculopathy	1.16 (1–1.35)	0.08	NA	NA	0.363	0	NA
Drugs affecting bone structure and mineralization	Diabetic retinopathy	1.11 (1.04–1.19)	0.01	NA	NA	0.86	0	NA
Drugs affecting bone structure and mineralization	Neovascular glaucoma	0.96 (0.78–1.18)	0.7	NA	NA	0.709	0	NA
Drugs affecting bone structure and mineralization	Primary open‐angle glaucoma	1 (0.93–1.08)	0.95	NA	NA	0.732	0	NA
Drugs affecting bone structure and mineralization	Senile cataract	1.03 (0.98–1.07)	0.29	NA	NA	0.352	0	NA
Drugs for peptic ulcer and gastro‐esophageal reflux disease (GERD)	Neovascular glaucoma	0.49 (0.22–1.08)	0.15	NA	NA	0.386	0	NA
Drugs for peptic ulcer and gastro‐esophageal reflux disease (GERD)	Primary open‐angle glaucoma	0.86 (0.6–1.24)	0.46	NA	NA	0.199	0	NA
Drugs for peptic ulcer and gastro‐esophageal reflux disease (GERD)	Senile cataract	1.04 (0.92–1.19)	0.55	NA	NA	0.527	0	NA
Drugs used in diabetes	Diabetic maculopathy	1.17 (1.1–1.25)	0	NA	NA	0.831	0	NA
Drugs used in diabetes	Diabetic retinopathy	1.08 (1.04–1.12)	0	NA	NA	0.861	0	NA
Drugs used in diabetes	Neovascular glaucoma	1.35 (1.2–1.52)	0	NA	NA	0.072	0	NA
Drugs used in diabetes	Primary open‐angle glaucoma	1.09 (1.04–1.15)	0	NA	NA	0.082	0	NA
Drugs used in diabetes	Senile cataract	1.08 (1.05–1.1)	0	NA	NA	0.094	0	NA
Glucocorticoids	Diabetic maculopathy	1.22 (1.05–1.41)	0.02	NA	NA	0.65	0	NA
Glucocorticoids	Diabetic retinopathy	1.23 (1.12–1.35)	0	NA	NA	0.631	0	NA
Glucocorticoids	Neovascular glaucoma	1.32 (1.07–1.63)	0.02	NA	NA	0.129	0	NA
Glucocorticoids	Primary open‐angle glaucoma	1.08 (1–1.17)	0.08	NA	NA	0.189	0	NA
Glucocorticoids	Senile cataract	1.08 (1.03–1.13)	0.01	NA	NA	0.07	0	NA
HMG CoA reductase inhibitors	Diabetic maculopathy	0.92 (0.85–1.01)	0.08	NA	NA	0.261	0	NA
HMG CoA reductase inhibitors	Diabetic retinopathy	0.88 (0.83–0.94)	0	NA	NA	0.082	0	NA
HMG CoA reductase inhibitors	Neovascular glaucoma	1.01 (0.87–1.18)	0.88	NA	NA	0.07	0	NA
HMG CoA reductase inhibitors	Primary open‐angle glaucoma	0.91 (0.86–0.97)	0	NA	NA	0.08	0	NA
HMG CoA reductase inhibitors	Senile cataract	0.99 (0.95–1.02)	0.55	NA	NA	<0.001	0	NA
Salicylic acid and derivatives	Diabetic maculopathy	0.95 (0.72–1.24)	0.7	NA	NA	0.373	0	NA
Salicylic acid and derivatives	Diabetic retinopathy	0.93 (0.79–1.09)	0.4	NA	NA	0.443	0	NA
Salicylic acid and derivatives	Neovascular glaucoma	0.66 (0.43–1)	0.08	NA	NA	0.543	0	NA
Salicylic acid and derivatives	Primary open‐angle glaucoma	0.83 (0.68–1.03)	0.13	NA	NA	0.235	0	NA
Salicylic acid and derivatives	Senile cataract	1.06 (0.96–1.17)	0.26	NA	NA	0.229	0	NA
Thyroid preparations	Diabetic maculopathy	1.2 (1.14–1.27)	0	NA	NA	0.044	0	NA
Thyroid preparations	Diabetic retinopathy	1.14 (1.09–1.19)	0	NA	NA	<0.001	0	NA
Thyroid preparations	Neovascular glaucoma	1.02 (0.94–1.11)	0.58	NA	NA	0.46	0	NA
Thyroid preparations	Primary open‐angle glaucoma	0.99 (0.96–1.03)	0.74	NA	NA	0.028	0	NA
Thyroid preparations	Senile cataract	1.05 (1.03–1.06)	0	NA	NA	0.204	0	NA

*Note:* Global *P* indicates pleiotropy. Outlier‐corrected OR and *P* show results after removing outlier SNPs.

**Table 2:** Heterogeneity and pleiotropy tests for the causal associations.

Exposure	Outcome	Heterogeneity		Pleotropy	
		*Q* statistic (IVW)	*p*‐Value	MR‐Egger intercept	*p*‐Value
Calcium channel blockers	Diabetic maculopathy	23.2117	0.3328	−0.0251	0.2117
	Neovascular glaucoma	19.2787	0.5038	−0.0431	0.3092
	Primary open‐angle glaucoma	20.3062	0.3158	0.0236	0.0748
	Diabetic retinopathy	22.426	0.3753	2.85E‐05	0.9981
	Senile cataract	32.0838	0.0574	0.013	0.0634
Beta blocking agents	Diabetic maculopathy	18.7246	0.1319	−0.0462	0.2685
	Neovascular glaucoma	11.127	0.6002	−0.0289	0.6185
	Primary open‐angle glaucoma	14.1388	0.3641	0.0048	0.8426
	Diabetic retinopathy	10.1631	0.4263	−0.0235	0.4457
	Senile cataract	15.8356	0.2581	0.0032	0.7869
Diuretics	Diabetic maculopathy	18.788	0.405	−0.015	0.3564
	Neovascular glaucoma	11.7491	0.7611	−0.1343	0.0516
	Primary open‐angle glaucoma	21.5481	0.2027	−0.0096	0.4083
	Diabetic retinopathy	25.3181	0.1164	−0.0167	0.132
	Senile cataract	19.4157	0.3667	−0.0013	0.7892
Drugs used in diabetes	Diabetic maculopathy	8.0729	0.5268	0.0406	0.1755
	Neovascular glaucoma	5.9167	0.5495	−0.003	0.9608
	Primary open‐angle glaucoma	13.223	0.1528	−0.0069	0.7623
	Diabetic retinopathy	8.8208	0.454	0.0089	0.6145
	Senile cataract	7.7939	0.555	−0.0124	0.1603
Adrenergics, inhalants	Diabetic maculopathy	15.908	0.1023	0.0063	0.8236
	Neovascular glaucoma	4.898	0.8979	−0.0206	0.5681
	Primary open‐angle glaucoma	7.645	0.3649	−0.0065	0.7586
	Diabetic retinopathy	11.0447	0.354	−0.0062	0.6574
	Senile cataract	8.131	0.3212	0.0041	0.6638
Thyroid preparations	Diabetic maculopathy	23.6535	0.2098	−0.0189	0.161
	Neovascular glaucoma	13.4453	0.815	−0.028	0.186
	Primary open‐angle glaucoma	16.7653	0.3331	0.0024	0.7948
	Diabetic retinopathy	24.6615	0.0547	0.0152	0.4299
	Senile cataract	12.9639	0.5294	−0.0136	0.0603
Agents acting on the renin–angiotensin system	Diabetic maculopathy	34.1992	0.6011	−0.0318	0.0623
	Neovascular glaucoma	44.9132	0.1742	−0.01	0.7499
	Primary open‐angle glaucoma	28.3381	0.8149	0.0027	0.8022
	Diabetic retinopathy	26.1731	0.7947	0.0025	0.8096
	Senile cataract	28.3168	0.6536	−0.0002	0.9632
HMG CoA reductase inhibitors	Diabetic maculopathy	12.2205	0.5097	0.0444	0.0604
	Neovascular glaucoma	12.5264	0.485	−0.0046	0.9015
	Primary open‐angle glaucoma	13.3363	0.4222	0.0195	0.1813
	Diabetic retinopathy	19.5947	0.1058	0.0256	0.1046
	Senile cataract	18.8845	0.1267	−0.0011	0.8936
Drugs affecting bone structure and mineralization	Diabetic maculopathy	1.9466	0.163	—	—
	Neovascular glaucoma	1.3699	0.2418	—	—
	Primary open‐angle glaucoma	1.1236	0.2891	—	—
	Diabetic retinopathy	0.445	0.5047	—	—
	Senile cataract	3.5457	0.0597	—	—
Glucocorticoids	Diabetic maculopathy	1.65E‐05	0.9968	—	—
	Neovascular glaucoma	0.043	0.8356	—	—
	Primary open‐angle glaucoma	0.0888	0.7657	—	—
	Diabetic retinopathy	0.3309	0.5651	—	—
	Senile cataract	0.085	0.7706	—	—
Antiglaucoma preparations and miotics	Diabetic maculopathy	1.441	0.4865	0.0483	0.7238
	Neovascular glaucoma	0.9484	0.6224	−0.1011	0.6434
	Diabetic retinopathy	0.6244	0.7319	0.0446	0.5805
	Senile cataract	15.8286	4e–04	0.1092	0.2053

*Note:* Heterogeneity was assessed by Cochran’s *Q* statistic. Horizontal pleiotropy was evaluated by the MR‐Egger intercept. *p*  < 0.05 indicates significance.

Post‐correction analysis revealed robust associations (P‐FDR < 0.05) for specific drug‐outcome pairs. Drugs used in diabetes showed significant associations with an increased risk of senile cataract. Similarly, adrenergics (inhalants) were significantly associated with an increased risk of senile cataract.

**Figure 2:**
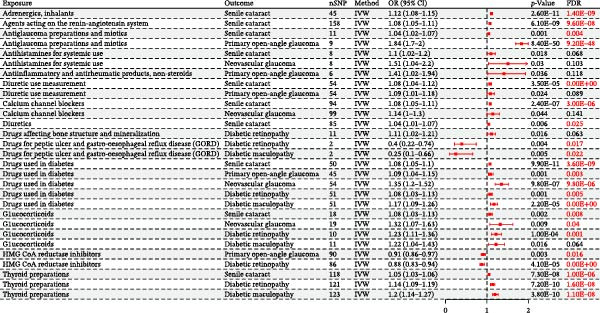
Forest plot comparing causal estimates from univariable Mendelian randomization (positive results).

Additionally, several other medications showed nominal significance (*p*  < 0.05) in the primary IVW analysis and were prioritized for subsequent validation. These included thyroid preparations (associated with DMac and DR), HMG CoA reductase inhibitors (associated with a reduced risk of DR), and beta‐blocking agents (associated with senile cataract) (Table 3 and Figure 2). Results for all analyzed associations are detailed in Supporting Information 1: Table S5–7.

**Table 3:** Causal associations between commonly used medications and diabetic eye diseases.

Exposure	Outcome	N.SNPs	Methods	OR (95% CI)	*p*	FDR
Adrenergics, inhalants	Senile cataract	45	IVW	1.12 (1.08–1.15)	2.60E − 11	1.40E − 09
Agents acting on the renin–angiotensin system	Senile cataract	158	IVW	1.08 (1.05–1.11)	6.10E − 09	9.60E − 08
Antiglaucoma preparations and miotics	Primary open‐angle glaucoma	9	IVW	1.84 (1.7–2)	8.40E − 50	9.20E − 48
Antiglaucoma preparations and miotics	Senile cataract	11	IVW	1.04 (1.02–1.07)	7.30E‐04	0.004
Antihistamines for systemic use	Neovascular glaucoma	8	IVW	1.51 (1.04–2.2)	0.03	0.103
Antihistamines for systemic use	Senile cataract	8	IVW	1.1 (1.02–1.2)	0.018	0.068
Antiinflammatory and antirheumatic products, nonsteroids	Primary open‐angle glaucoma	6	IVW	1.41 (1.02–1.94)	0.036	0.118
Antimigraine preparations	Primary open‐angle glaucoma	13	IVW	1.06 (1–1.12)	0.07	0.197
Diuretic use measurement	Primary open‐angle glaucoma	54	IVW	1.09 (1.01–1.18)	0.024	0.089
Diuretic use measurement	Senile cataract	54	IVW	1.08 (1.04–1.12)	3.50E − 05	2.80E − 04
Calcium channel blockers	Neovascular glaucoma	99	IVW	1.14 (1–1.3)	0.044	0.141
Calcium channel blockers	Senile cataract	94	IVW	1.08 (1.05–1.11)	2.40E − 07	3.00E − 06
Diuretics	Senile cataract	85	IVW	1.04 (1.01–1.07)	0.006	0.025
Drugs affecting bone structure and mineralization	Diabetic retinopathy	11	IVW	1.11 (1.02–1.21)	0.016	0.063
Drugs for peptic ulcer and gastro‐esophageal reflux disease (GERD)	Diabetic maculopathy	2	IVW	0.25 (0.1–0.66)	0.005	0.022
Drugs for peptic ulcer and gastro‐esophageal reflux disease (GERD)	Diabetic retinopathy	2	IVW	0.4 (0.22–0.74)	0.004	0.017
Drugs used in diabetes	Diabetic maculopathy	51	IVW	1.17 (1.09–1.26)	2.20E − 05	2.00E − 04
Drugs used in diabetes	Neovascular glaucoma	54	IVW	1.35 (1.2–1.52)	9.80E − 07	9.80E − 06
Drugs used in diabetes	Primary open‐angle glaucoma	45	IVW	1.09 (1.04–1.15)	5.60E − 04	0.003
Drugs used in diabetes	Diabetic retinopathy	51	IVW	1.08 (1.03–1.13)	8.70E − 04	0.005
Drugs used in diabetes	Senile cataract	50	IVW	1.08 (1.05–1.1)	9.90E − 11	3.60E − 09
Glucocorticoids	Diabetic maculopathy	11	IVW	1.22 (1.04–1.43)	0.016	0.064
Glucocorticoids	Neovascular glaucoma	19	IVW	1.32 (1.07–1.63)	0.009	0.04
Glucocorticoids	Diabetic retinopathy	10	IVW	1.23 (1.11–1.36)	1.00E − 04	6.70E − 04
Glucocorticoids	Senile cataract	18	IVW	1.08 (1.03–1.13)	0.002	0.008
HMG CoA reductase inhibitors	Primary open‐angle glaucoma	90	IVW	0.91 (0.86–0.97)	0.003	0.016
HMG CoA reductase inhibitors	Diabetic retinopathy	86	IVW	0.88 (0.83–0.94)	4.10E − 05	2.80E − 04
Thyroid preparations	Diabetic maculopathy	123	IVW	1.2 (1.14–1.27)	3.80E − 10	1.10E − 08
Thyroid preparations	Diabetic retinopathy	121	IVW	1.14 (1.09–1.19)	7.20E − 10	1.60E − 08
Thyroid preparations	Senile cataract	118	IVW	1.05 (1.03–1.06)	7.30E − 08	1.00E − 06

*Note:* Results based on the Inverse Variance Weighted (IVW) method. *p*  < 0.05 indicates statistical significance.

Abbreviations: CI, confidence interval; FDR, false discovery rate; OR, odds ratio.

### 3.3. MVMR: Associations Independent of Key Confounding Factors

To distinguish whether the associations observed in the univariable analysis were driven by direct pharmacological effects or confounded by the underlying indications, we performed a multivariable Mendelian randomization (MVMR) analysis adjusting for key genetic confounders derived from the MVP dataset (Supporting Information 2: Table S8, Supporting Information 3: Table S9). Crucially, several associations remained robust after adjusting for their respective primary indications.

**Figure 3:**
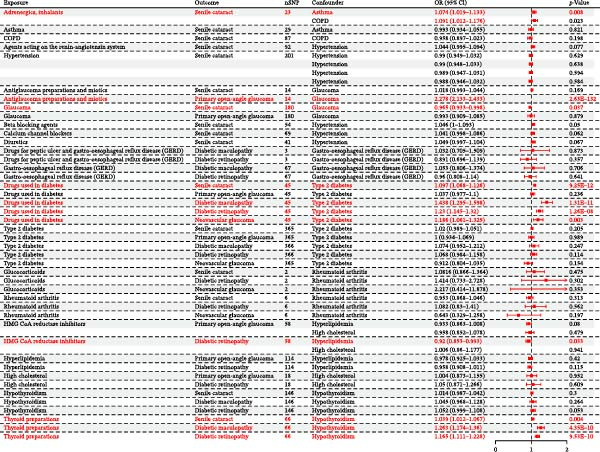
Forest plot comparing causal estimates from MVMR Results.

Analysis results indicate that after adjusting for asthma and COPD, the causal association between adrenergics (inhalants) and senile cataract (asthma: OR = 1.074, 95% CI: 1.019–1.133, *p* = 0.008; COPD: OR = 1.091, 95% CI: 1.012–1.176, *p* = 0.023) remains statistically significant; after adjusting for glaucoma, the causal association between antiglaucoma preparations and miotics with POAG (OR = 2.278, 95% CI: 2.133–2.433, *p* = 2.63 × 10^−132^) remains statistically significant; after adjusting for hypertension, the causal association between beta‐blocking agents and senile cataract (OR = 1.046, 95% CI: 1.000–1.093, *p* = 0.050) remained statistically significant; after adjusting for type 2 diabetes, the causal associations between drugs used in diabetes with DMac (OR = 1.438, 95% CI: 1.295–1.598, *p* = 1.31 × 10^−11^), DR (OR = 1.230, 95% CI: 1.145–1.320, *p* = 1.26 × 10^−8^), NVG (OR = 1.186, 95% CI: 1.061–1.325, *p* = 0.003), and senile cataract (OR = 1.097, 95% CI: 1.068–1.126, *p* = 9.25 × 10^−12^) remained statistically significant; after adjusting for hyperlipidemia, the causal association between HMG CoA reductase inhibitors and DR (OR = 0.920, 95% CI: 0.853–0.993, *p* = 0.033) remained statistically significant; and after adjusting for hypothyroidism, the causal association between thyroid preparations with DMac (OR = 1.263, 95% CI: 1.174–1.360, *p* = 4.35 × 10^−10^), DR (OR = 1.168, 95% CI: 1.111–1.228, *p* = 9.53 × 10^−10^), and senile cataract (OR = 1.039, 95% CI: 1.012–1.067, *p* = 0.004) remained statistically significant (Table 4 and Figure 3).

**Table 4:** Multivariable Mendelian randomization (MVMR) analysis adjusted for confounders.

Exposure	Outcome	N.SNP	Adjusted for	OR (95% CI)	*p*‐Value
Adrenergics, inhalants	Senile cataract	23	Asthma	1.074 (1.019–1.133)	0.008
		13	COPD	1.091 (1.012–1.176)	0.023
Agents acting on the renin–angiotensin system	Senile cataract	92	Hypertension	1.044 (0.995–1.094)	0.077
Antiglaucoma preparations and miotics	Primary open‐angle glaucoma	14	Glaucoma	2.278 (2.133–2.433)	2.63E − 132
Antiglaucoma preparations and miotics	Senile cataract	14	Glaucoma	1.018 (0.993–1.044)	0.169
Beta blocking agents	Senile cataract	54	Hypertension	1.046 (1.000–1.093)	0.05
Calcium channel blockers	Senile cataract	69	Hypertension	1.041 (0.998–1.086)	0.062
Diuretics	Senile cataract	41	Hypertension	1.049 (0.997–1.104)	0.067
Drugs for peptic ulcer and gastro‐esophageal reflux disease (GERD)	Diabetic maculopathy	3	Gastro‐esophageal reflux disease (GERD)	1.032 (0.705–1.509)	0.873
Drugs for peptic ulcer and gastro‐esophageal reflux disease (GERD)	Diabetic retinopathy	3	Gastro‐esophageal reflux disease (GERD)	0.891 (0.696–1.139)	0.357
Drugs used in diabetes	Diabetic maculopathy	45	Type 2 diabetes	1.438 (1.295–1.598)	1.31E − 11
Drugs used in diabetes	Diabetic retinopathy	45	Type 2 diabetes	1.230 (1.145–1.320)	1.26E − 08
Drugs used in diabetes	Neovascular glaucoma	45	Type 2 diabetes	1.186 (1.061–1.325)	0.003
Drugs used in diabetes	Primary open‐angle glaucoma	45	Type 2 diabetes	1.037 (0.977–1.100)	0.236
Drugs used in diabetes	Senile cataract	45	Type 2 diabetes	1.097 (1.068–1.126)	9.25E‐12
Glucocorticoids	Diabetic retinopathy	2	Rheumatoid arthritis	1.414 (0.733–2.728)	0.302
Glucocorticoids	Neovascular glaucoma	2	Rheumatoid arthritis	2.217 (0.414–11.878)	0.353
Glucocorticoids	Senile cataract	2	Rheumatoid arthritis	1.086 (0.866–1.364)	0.475
HMG CoA reductase inhibitors	Diabetic retinopathy	24	High cholesterol	1.006 (0.860–1.177)	0.941
		58	Hyperlipidemia	0.920 (0.853–0.993)	0.033
HMG CoA reductase inhibitors	Primary open‐angle glaucoma	24	High cholesterol	0.958 (0.852–1.078)	0.479
		58	Hyperlipidemia	0.933 (0.863–1.008)	0.08
Thyroid preparations	Diabetic maculopathy	66	Hypothyroidism	1.263 (1.174–1.360)	4.35E‐10
Thyroid preparations	Diabetic retinopathy	66	Hypothyroidism	1.168 (1.111–1.228)	9.53E‐10
Thyroid preparations	Senile cataract	66	Hypothyroidism	1.039 (1.012–1.067)	0.004

*Note:* Estimates adjusted for key confounders (e.g., hypertension, type 2 diabetes, and asthma). *p*  < 0.05 indicates an independent causal effect.

However, when we adjusted for other noncorresponding confounders, both associations were attenuated to nonsignificance (all *p*  > 0.05). For example, after adjusting for rheumatoid arthritis, the associations between glucocorticoids and DR (OR = 1.414, 95% CI: 0.733–2.728, *p* = 0.302), NVG (OR = 2.217, 95% CI: 0.414–11.878, *p* = 0.353), and senile cataract (OR = 1.086, 95% CI: 0.866–1.364, *p* = 0.475) were no longer significant (Table 4 and Figure 3).

### 3.4. Gene Mapping

As common medications for diabetes‐related eye diseases, the gene mapping process focused on drugs used in diabetes and agents acting on the renin–angiotensin system. The gene mapping results revealed 10 and 38 SNP annotations for drugs used in diabetes and agents acting on the renin–angiotensin system, respectively (Supporting Information 4: Table S10).

### 3.5. Functional Enrichment Analysis of Key Drugs

Key targets were identified based on SNP annotations for drugs used in diabetes and agents acting on the renin–angiotensin system. GO and KEGG functional enrichment analyses were performed on these key targets, selecting the top 10 pathways with *p*.adjust < 0.05. The pathways most enriched for targets associated with drugs used in diabetes are primarily hormone transport and adherens junction pathways. The pathways most enriched for targets associated with agents acting on the renin–angiotensin system are primarily regulation of lyase activity and the MAPK signaling pathway (Figure 4).

Figure 4GO/KEGG Pathway Enrichment Results. (A,B): GO/KEGG Pathway Enrichment Results of Drugs used in diabetes; (C,D): GO/KEGG Pathway Enrichment Results of Agents acting on the renin–angiotensin system.

**Figure:**
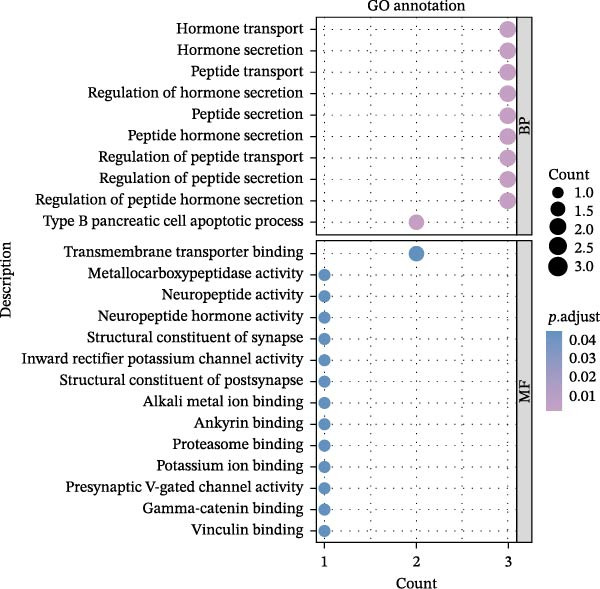
(A)

**Figure:**
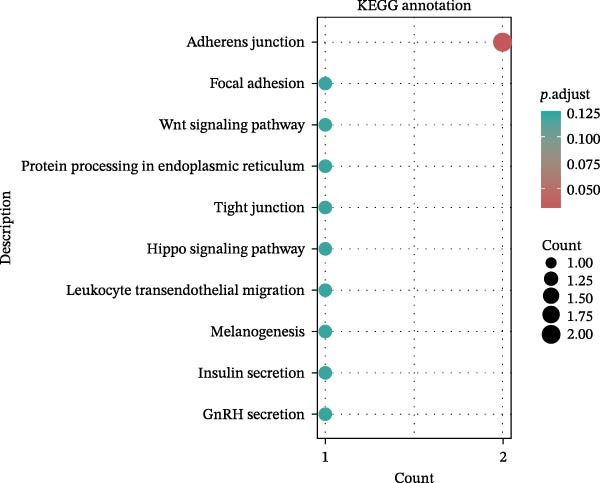
(B)

**Figure:**
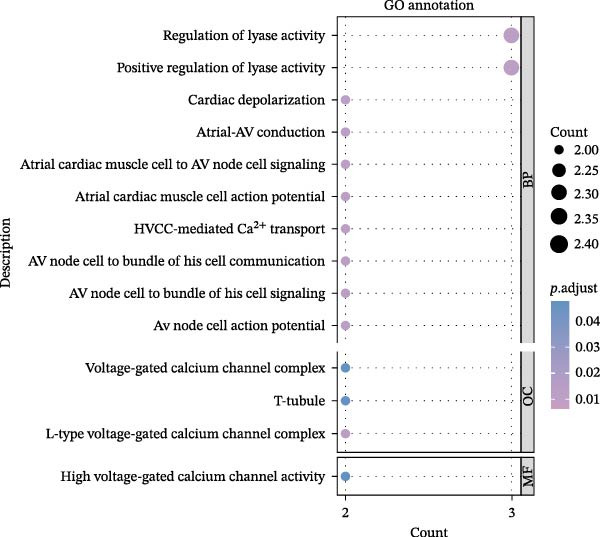
(C)

**Figure:**
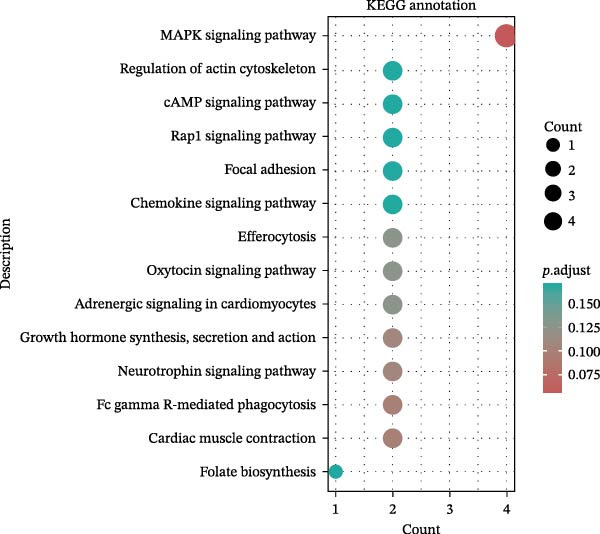
(D)

## 4. Discussion

In this comprehensive MR study, we identified and validated multiple robust associations between genetically predicted use of common medications and risk of diabetic eye diseases. While initial MR analyses revealed numerous positive signals, MVMR, adjusting for key comorbidities such as type 2 diabetes, hypertension, hyperlipidemia, hypothyroidism, asthma/COPD, and glaucoma, confirmed that only a subset of these associations remained independent, including links between adrenergic inhalants, beta‐blockers, antiglaucoma agents, diabetes medications, statins, and thyroid preparations with specific ocular outcomes. Notably, gene mapping prioritized drugs used in diabetes and agents acting on the renin–angiotensin system as exposure categories with strong genomic anchoring, and subsequent pathway enrichment implicated biologically coherent mechanisms: hormone transport and adherens junction pathways for diabetes drugs and regulation of lyase activity and MAPK signaling for renin–angiotensin system agents. Together, these integrated genetic, epidemiological, and functional findings provide a prioritized, mechanistically informed framework for understanding how systemic pharmacotherapies may influence the development or progression of diabetic eye complications.

MR analysis revealed several significant correlations between genetically predicted drug exposure and diabetic eye disease. After adjusting for confounding factors and applying FDR correction, we still observed an association between inhaled adrenergic medications and senile cataract. The potential biological mechanisms linking inhaled adrenergic medications (such as β2‐adrenergic agonists like salbutamol) to cataract formation may involve multiple pathways. Firstly, oxidative stress constitutes a central mechanism in cataract development. β2‐adrenergic agonists may activate β2‐adrenergic receptors on lens epithelial cells (LECs), leading to elevated intracellular cyclic adenosine monophosphate (cAMP) levels [40] and subsequent activation of protein kinase A (PKA) [41]. This signaling pathway may upregulate NADPH oxidase activity, promoting reactive oxygen species (ROS) production. This leads to oxidative damage and aggregation of lens proteins, ultimately resulting in opacity formation. Secondly, osmotic imbalance represents another critical factor. Research suggests β2 agonists may affect the function of ionic pumps (e.g., Na^+^/K^+^‐ATPase) [42] or the expression of aquaporins within [43] the lens, leading to water accumulation and osmotic imbalance in lens fiber cells, thereby accelerating lens opacity. Furthermore, disruption of glucose metabolism should not be overlooked. Prolonged β2‐receptor stimulation may activate the sorbitol pathway and cause polyol accumulation within the lens by affecting insulin signaling [44] pathways or directly acting on lens glucose metabolism enzymes, thereby inducing osmotic swelling and damage.

Additionally, other potential connections exist. Specifically, we observed positive signals linking antiglaucoma preparations and miotics to an increased risk of POAG, a finding that, at face value, appears counterintuitive given their therapeutic intent; drugs used in diabetes showed broad associations with multiple ocular complications, including DR, DMac, NVG, and senile cataract, suggesting a systemic influence beyond glycemic control; HMG CoA reductase inhibitors (statins) were linked to a reduced risk of DR, aligning with prior epidemiological and experimental evidence of their anti‐inflammatory and endothelial‐protective properties [45, 46]; and thyroid preparations demonstrated associations with DR, DMac, and senile cataract, hinting at a potential role for thyroid hormone signaling in ocular tissue homeostasis.

Collectively, these preliminary signals generate a wide‐ranging set of hypotheses about how commonly prescribed medications might modulate the risk or progression of sight‐threatening complications in individuals with diabetes. However, interpreting these associations requires caution. Medication use is inherently tied to underlying clinical indications, such as hypertension for beta‐blockers, dyslipidemia for statins, or hypothyroidism for thyroid hormone replacement, and these conditions themselves are established or suspected risk factors for various eye diseases. For instance, long‐standing diabetes drives both the need for glucose‐lowering drugs and the development of microvascular complications [47]; similarly, chronic glaucoma may lead to intensified use of antiglaucoma agents while also progressing independently. As a result, the observed MR estimates in the univariable setting may reflect confounding by indication, disease severity, or shared genetic liability between the indication and the ocular outcome, rather than a direct pharmacological effect of the drug itself. This limitation underscores the necessity of multivariable approaches that explicitly account for these interrelated clinical phenotypes, a strategy we implemented in subsequent analyses to isolate more credible drug‐specific signals.

To disentangle true drug‐related effects from indication bias, we conducted MVMR adjusting for key clinical covariates. This more stringent approach revealed that only a subset of associations remained robust: (1) antiglaucoma preparations and miotics retained a positive association with POAG after adjustment for baseline glaucoma status, suggesting either a disease‐severity confounding pattern or a paradoxical biological effect of chronic miotic use; (2) beta‐blocking agents remained linked to senile cataract after accounting for hypertension, pointing to a potential off‐target ocular impact independent of blood pressure control; (3) drugs used in diabetes showed persistent associations with DR, DMac, NVG, and senile cataract even after adjusting for genetically predicted liability to type 2 diabetes, implying that specific glucose‐lowering therapies, or their systemic metabolic consequences, may modulate ocular tissue susceptibility beyond glycemic exposure alone; (4) statins were associated with lower DR risk after controlling for hyperlipidemia, supporting a plausible pleiotropic protective role in retinal microcirculation; and (5) thyroid preparations remained associated with DR, DMac, and senile cataract after adjustment for hypothyroidism, raising the possibility that thyroid hormone levels within the normal range may still influence ocular homeostasis. All other initially positive signals became nonsignificant in MVMR, underscoring the critical role of comorbidity adjustment in pharmacological MR studies.

To further assess biological plausibility and respond to the need for mechanistic insight, we performed gene mapping of drug‐associated SNPs and subsequent pathway enrichment analyses. This prioritized two therapeutically relevant categories: drugs used in diabetes and agents acting on the renin–angiotensin system. Notably, targets linked to diabetes drugs (e.g., KCNJ11, TCF7L2) were significantly enriched in hormone transport and adherens junction pathways. The integrity of adherens junctions is critical for the blood‐retinal barrier; its disruption is a hallmark of early DR and macular edema, suggesting that these medications may exert protective effects by stabilizing retinal vascular permeability beyond simple glycemic control [48–51]. Meanwhile, instruments for agents acting on the renin–angiotensin system mapped to the regulation of lyase activity and the MAPK signaling pathway. Although the causal association for RAS agents was attenuated in our MVMR analysis after adjusting for hypertension (*p* = 0.077), the enrichment in MAPK signaling—a central driver of oxidative stress and extracellular matrix remodeling in ocular fibrosis—highlights a plausible biological axis [52, 53]. These findings provide a mechanistically coherent framework that complements our epidemiological associations, offering prioritized targets for future experimental validation.

To our knowledge, this study is the first two‐sample MR, investigation of commonly used medications and diabetes‐related eye diseases. By using strong genetic instruments (*F*‐statistic ≥ 10) and large‐scale genomic data, our design minimizes confounding and reverse causality. We further strengthened causal inference through MVMR adjusting for key comorbidities, and enhanced biological interpretability via gene mapping and pathway enrichment, highlighting plausible mechanisms such as hormone transport, adherens junctions, and MAPK signaling. This integrated approach provides a more robust and mechanistically informed assessment of drug‐eye disease relationships.

This study has several important advantages. It is the first two‐sample MR study to examine the causal relationship between commonly used medication use and diabetes‐related eye diseases, and it most closely resembles an RCT by allowing random assignment based on genotype. This study design avoids some of the limitations of traditional observational studies, including reverse causality and potential confounders. Our MR study utilized the large sample size of the included studies as well as IVs (*F*‐statistic ≥ 10) that were strongly associated with commonly used medicines. Several limitations of this study warrant consideration. First, following the application of stringent IV selection criteria (*p*  < 5 × 10^−8^), fewer than three independent genetic variants were identified for several exposure traits, precluding the use of MR‐Egger regression to assess directional pleiotropy. Second, while our MVMR analysis rigorously adjusted for key confounding comorbidities (e.g., type 2 diabetes, hypertension), we acknowledge that genetic instruments for medication use may still capture elements of disease severity (indication bias) that are not fully corrected by binary disease traits. However, the persistence of associations—such as diabetes drugs with cataracts and retinopathy—even after adjusting for genetic liability to type 2 diabetes, suggests a potential drug‐specific effect beyond genetic liability to the underlying disease. Third, our exposure instruments were derived from the UK Biobank GWAS of self‐reported medication use (2006–2010), which may be subject to recall bias and cannot capture critical dimensions of drug exposure such as dose, duration, adherence, or formulation, limiting inference on adverse effects or dose–response relationships. Additionally, for some drug classes, the limited number of available SNPs restricted statistical power. However, for our primary findings, diabetes medications and inhaled adrenergics, both significant at the FDR level, we had sufficient instruments (>10 SNPs each) and conducted comprehensive sensitivity analyses (MR‐Egger and MR‐PRESSO), which showed no evidence of horizontal pleiotropy, supporting the robustness of these core associations. Furthermore, all genetic data were derived from individuals of European ancestry, which may limit the generalizability of our findings to other populations due to differences in allele frequencies, LD patterns, and medication utilization. Finally, although our multivariate MR analysis has adjusted for several key confounding factors, this method is inherently restricted to factors with reliable genetic instruments available from existing GWAS. Consequently, residual confounding effects from unmeasured or inadequately instrumented variables, such as environmental exposures, cannot be entirely ruled out, as these factors may influence the observed associations.

It is important to clarify a common point of confusion regarding exposure measurement in pharmacological MR. Although the genetic instruments used in this study were derived from a GWAS based on single‐timepoint, self‐reported medication use in the UK Biobank, which indeed lacks detail on dosage, duration, adherence, and long‐term patterns, the causal inference in MR does not depend on accurately capturing actual drug intake at one moment in time [54]. Instead, MR leverages genetic variants as lifelong, fixed proxies for an individual’s inherent biological propensity to use a given medication. Because these variants are randomly assigned at conception and remain stable across the lifespan, they reflect a cumulative tendency toward exposure, aligning more closely with the chronic, long‐term nature of conditions like diabetic eye disease than transient self‐reports ever could. In this way, MR inherently mitigates recall bias and cross‐sectional misclassification that plague conventional observational studies [55]. While this framework cannot support fine‐grained dose–response analyses or distinguish between drug formulations, it is well‐suited to test whether a sustained genetic predisposition to using a medication is associated with altered disease risk, providing a robust approximation of long‐term causal effects under the core MR assumptions.

Furthermore, we acknowledge that utilizing genetic variants in specific drug‐metabolizing enzymes would act as ideal instruments to validate direct pharmacological effects. However, due to the limited availability of large‐scale GWAS data for these specific enzymatic targets, this study relied on self‐reported medication usage. Consequently, we explicitly state this as a limitation, as it precludes the use of PheWAS to definitively rule out all potential sources of indication bias.

In summary, this MR study identifies several medication classes, particularly diabetes drugs, thyroid preparations, statins, beta‐blockers, and antiglaucoma agents, with robust, comorbidity‐adjusted associations with diabetic eye diseases. Integrated gene mapping and pathway analyses further implicate biologically plausible mechanisms, including hormone transport, adherens junction integrity, and MAPK signaling. While these findings do not establish clinical causality, they provide a prioritized, genetically informed framework for future experimental validation and pharmacoepidemiological investigation into the ocular effects of systemic medications in patients with diabetes.

## Author Contributions

Yu Wang, Mingyue Jin, Lamusi A., and Chak Kwong Cheng carried out the studies, participated in collecting data, and drafted the manuscript. Yu Wang and Chang Wang performed the statistical analysis and participated in its design. Mingyue Jin and Chang Wang participated in acquisition, analysis, or interpretation of data and draft the manuscript.

## Funding

This research was funded by the Shenzhen Overseas High‐Level Talent (Peacock Plan) Project Research Initiation Programme (Grant 827000745), the Shenzhen Medical Research Fund (Grant A2503054), and the HaiYa Young Scientist Foundation of Shenzhen University General Hospital (Grant 2025‐HY003).

## Disclosure

All authors read and approved the final manuscript.

## Ethics Statement

The authors have nothing to report.

## Consent

The authors have nothing to report.

## Conflicts of Interest

The authors declare no conflicts of interest.

## Supporting Information

Additional supporting information can be found online in the Supporting Information section.

## Supporting information


**Supporting Information 1** Table S1: The GWAS data for exposure and outcomes. Table S2: *F*‐statistical values for factor‐related IVs. Table S3: Detailed table of extracted SNPs and outcome matches. Table S4: Eliminated MR‐PRESSO results. Table S5: Tests for horizontal pleiotropy and heterogeneity after excluding outliers. Table S6: Excluded SNPs. Table S7: The relationship between commonly used medications and diabetic eye diseases after eliminating outliers (All results).

**Supporting Information 2** Table S8: Exposure‐outcome corresponding confounding factors.

**Supporting Information 3** Table S9: Detailed GWAS data for confounding factors.

**Supporting Information 4** Table S10: SNP gene annotation.

## Data Availability

All data generated or analyzed during this study are included in this published article and its supporting information files.
